# Hemoglobin Constant Spring among Southeast Asian Populations: Haplotypic Heterogeneities and Phylogenetic Analysis

**DOI:** 10.1371/journal.pone.0145230

**Published:** 2015-12-18

**Authors:** Wittaya Jomoui, Goonnapa Fucharoen, Kanokwan Sanchaisuriya, Van Hoa Nguyen, Supan Fucharoen

**Affiliations:** 1 Biomedical Science Program, Graduate School, Khon Kaen University, Khon Kaen, Thailand; 2 Centre for Research and Development of Medical Diagnostic Laboratories, Faculty of Associated Medical Sciences, Khon Kaen University, Khon Kaen, Thailand; 3 Hue Colleges of Medicine and Pharmacy, Hue, Vietnam; University of Naples Federico II, ITALY

## Abstract

**Background:**

Hemoglobin Constant Spring (Hb CS) is an abnormal Hb caused by a mutation at the termination codon of α2-globin gene found commonly among Southeast Asian and Chinese people. Association of Hb CS with α°-thalassemia leads to a thalassemia intermedia syndrome commonly encountered in the region. We report chromosome background and addressed genetic origins of Hb CS observed in a large cohort of Hb CS among Southeast Asian populations.

**Materials and Methods:**

A study was done on 102 Vietnamese (aged 15–49 year-old) and 40 Laotian (aged 18–39 year-old) subjects with Hb CS and results compared with 120 Hb CS genes in Thailand. Hematological parameters were recorded and Hb analysis was performed using capillary electrophoresis. Hb CS mutation and thalassemia genotypes were defined by DNA analysis. Six DNA polymorphisms within α-globin gene cluster including 5’*Xba* I, *Bgl* I, Inter-zeta HVR, *Acc*I, *RsaI* and α*Pst*I 3’, were determined using PCR-RFLP assay.

**Results:**

Nine different genotypes of Hb CS were observed. In contrast to the Thai Hb CS alleles which are mostly linked to haplotype (+—S + + -), most of the Vietnamese and the Laotian Hb CS genes were associated with haplotype (+—M + + -), both of which are different from that of the European Hb CS.

**Conclusions:**

Hb CS is commonly found in combination with other thalassemias among Southeast Asian populations. Accurate genotyping of the cases requires both hematologic and DNA analyses. At least two independent origins are associated with the Hb CS gene which could indirectly explain the high prevalence of this Hb variant in the region.

## Introduction

Hemoglobin Constant Spring (Hb CS) is an abnormal Hb caused by a mutation at the termination codon of an α2-globin gene, *T*AA—*C*AA. This mutation leads to the synthesis of unstable and elongated α-globin chains with 172 instead of 141 amino acid residues [1]. The mRNA of the Hb CS gene is very unstable. The level of Hb CS in the peripheral blood is very small and might be difficult to detect. An amount of 1–2% of total Hb is usually observed in a heterozygote. Homozygosity of Hb CS could be associated with a thalassemia intermedia phenotype with mild anemia, jaundice and hepatosplenomegaly [2]. Association of Hb CS with α^0^-thalassemia can lead to severe Hb H disease commonly encountered in China and Southeast Asia [3–6]. Although Hb CS is found mainly in China and Southeast Asia, it has been sporadically reported in the Mediterranean and Middle East regions [7]. We have recently documented the highest prevalence of Hb CS in the Có-Tu ethnic minority in central Vietnam; no haplotype information is available for tracing of background and origin of this variant gene [8]. Preliminary analysis of α-globin gene haplotypes associated with Hb CS genes in Chinese and Mediterranean patients pointed to different origins of this Hb variant [9]. This has been confirmed in our study of Thai patients with Hb CS in which at least two different major haplotypes have been noted [10]. Vietnam, a country located in Southeast Asia, has its borders in close contacts with China and Lao PDR in the northern parts and Cambodia in the southern parts of the country. To study the molecular heterogeneity of Hb CS and provide population genetics information of this Hb variant in the region, we have studied hematological phenotypes of several Hb CS related disorders, and examined the Hb CS linked haplotypes in this ethnic minority of Vietnam and Laos. Results were compared with available data from other populations.

## Materials and Methods

### Subjects and hematological analysis

Ethical approval of the study protocol was obtained from the Institutional Review Board (IRB) of Khon Kaen University, Thailand (HE562275). Since the current study was done on anonymous leftover DNA specimens from our previous projects which have been approved by the IRB of Khon Kaen University i.e. Implementation of thalassemia prevention and control program in Maria Teresa Hospital, Vientiane Lao People’s Democratic Republic (HE551414) and Thalassemia and iron Deficiency in Thua Thien Hue, Vietnam (HE552103), the IRB of Khon Kaen University waived the need for consent in the current study. A total of 102 Vietnamese DNA specimens were selectively recruited from our previous studies [8,11]. They were unrelated Có-Tu ethnic women (aged 15–49 years) inhabiting Thua Thien Hue province in the central region of Vietnam. All subjects were apparently healthy, based on a physical examination and anthropometric measurement, and had no history or symptoms of inflammatory diseases. Those with multi-parity, currently on iron medication and with chronic inflammatory diseases were excluded. Additional DNA specimens were also obtained from Laotian pregnant women (aged 18–39 years) who were attending antenatal care units at hospitals in Vientiane, Laos PDR, described in our earlier study (n = 40) [12]. All of them carried Hb CS with various genotypes. Hematological parameters were recorded on the KX-21 automated blood cell counter (Sysmex, Kobe, Japan). Hemoglobin analysis was done using automated capillary electrophoresis (Capillarys2 Flex Piecing, Sebia, Lisses, France).

### DNA analysis

Hb CS mutation was confirmed using allele specific polymerase chain reaction (PCR) as described previously [6, 13]. Identifications of other common α-thalassemia including α^0^-thalassemia (SEA *&* THAI deletions) and α^+^-thalassemia 2 (-α^3.7^ & -α^4.2^) were performed routinely in our laboratory using the PCR methods described elsewhere [14, 15]. Only subjects with the Hb CS gene were subjected to α-globin gene haplotype analysis as described [10, 16]. Six DNA polymorphisms in the α-globin gene cluster including *Xba*I, *Sac*I, *Bgl*I, *Acc*I, *RsaI*, and α *Pst*I were determined using PCR-RFLP based assays. Hb CS associated haplotypes were segregated only from those of homozygous Hb CS and heterozygous Hb CS but alternatively with homozygous genotype for polymorphic pattern.

### Phylogenetic analysis

A dendrogram including all haplotypes observed was used to construct a phylogenetic tree with DendroUPGMA software (http://genomes.urv.cat/UPGMA/), applying Jaccard (Tanimoto) coefficient with default settings and 100 bootstrap replicates. The FigTree v.1.4.0 software was then used to design a graphical viewer of the phylogenetic tree.

### Statistical analysis

Mean and standard deviation were used to describe all hematological parameters of the subjects and p-value was generated using One Way ANOVA test. Statistical analysis was performed using the MINITAB release 14.12.0 statistical software. A statistically significant difference was considered as a p-value of less than 0.05.

## Results

Identification of the Hb CS mutation on the α2-globin gene using allele specific PCR confirmed the *T*AA—*C*AA mutation in all 102 Vietnamese and 40 Laotian subjects recruited. However, complete hematological parameters could only be obtained from a total of 130 cases. DNA analysis of these cases identified nine different Hb CS genotypes, including heterozygous Hb CS (α^CS^ α/ αα, β^A^/β^A^, n = 89), double heterozygous Hb CS/Hb E (α^CS^ α/α, β^A^/β^E^, n = 23), homozygous Hb CS (α^CS^ α/ α^CS^α, β^A^/β^A^, n = 9), compound heterozygous Hb CS/α^+^-thalassemia (α^CS^α/–α^3.7^, β^A^/ β^A^) (n = 3), Hb CS-Hb H disease (α^CS^ α/––^SEA^, β^A^/ β^A^) (n = 2) and four other rare genotypes found with a single case each. Hematological findings of these Hb CS genotypes are summarized in **Table 1**. It was found that subjects with the two most common genotypes i.e. Hb CS heterozygote or double heterozygote Hb CS/Hb E, had relatively normal hematological findings with a very mild anemia. However, more pronounced hematological changes were observed with other genotypes.

**Table 1:** Hematological parameters and genotypes of Vietnamese and Laotian subjects with Hb CS (n = 130). Data are presented as mean ± standard deviation or as raw data where appropriate. Nd: not determined.

Genotypes	No.	Rbc (x10^12^)	Hb (g/dl)	Hct (%)	MCV (fl)	MCH (pg)	MCHC (g/dl)	RDW (%)	Hb A_2_ (%)
α^CS^α/αα; β^A^/β^A^	89	4.7±0.5	12.0±1.3	38.6±5.8	83.3±6.1	25.7±2.1	30.8±1.8	14.2±1.5	2.1±0.2
α^CS^α/αα; β^A^/β^E^	23	4.6±0.5	11.4±1.4	37.2±4.3	80.2±4.2	24.6±1.8	30.6±2.1	14.6±1.8	3.2±0.5 ^a^
α^CS^α/α^CS^α; β^A^/β^A^	9	4.2±0.3	9.9±0.8	34.6±3.8	82.8±8.5	23.8±2.1	28.8±1.9	16.4±1.1	1.4±0.2^a^
α^CS^α/-α^3.7^; β^A^/β^A^	3	4.6±0.5	10.1±0.8	34.5±3.6	75.5±0.9	22.3±1.2	29.5±1.5	15.7±2.1	2.0±0.3
α^CS^α/—^SEA^; β^A^/β^A^	2	6.2, 3.6	11.3, 6.8	44.6, 24.5	71.0, 68.0	18.1, 18.7	25.4, 27.8	22.2, 22.9	0.4, 0.6
α^CS^α/αα; β^0^/β^E^	1	5.0	8.7	29.1	59.0	17.6	30.0	16.5	Nd
α^CS^α/-α^3.7^; β^A^/β^E^	1	4.1	8.9	30.1	74.0	22.0	29.5	17.8	Nd
α^CS^α/α^CS^α; β^A^/β^E^	1	3.9	7.9	30.7	78.0	20.1	25.6	16.0	Nd
α^CS^α/αα; β^0^/β^A^	1	5.3	11.4	37.7	70.8	21.4	30.2	13.8	4.7

^a^: Significant difference from the corresponding value of Hb CS heterozygote (α^CS^α/αα; β^A^/β^A^) at *P* < 0.05 using one-way ANOVA test.

**Fig 1:**
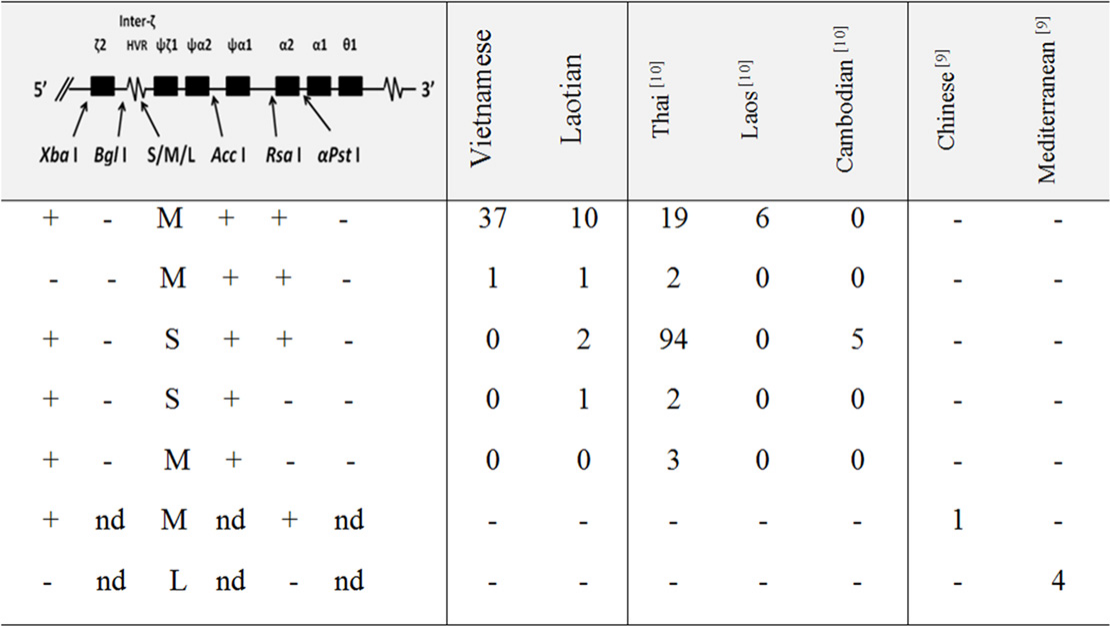
α-Globin gene haplotypes associated with Hb CS genes in Vietnamese and Lao subjects as compared to those described in other populations. Number of alleles detected in each haplotype is indicated. Plus and minus indicate the presence and absence of each polymorphic site. nd: not determined.

**Fig 2:**
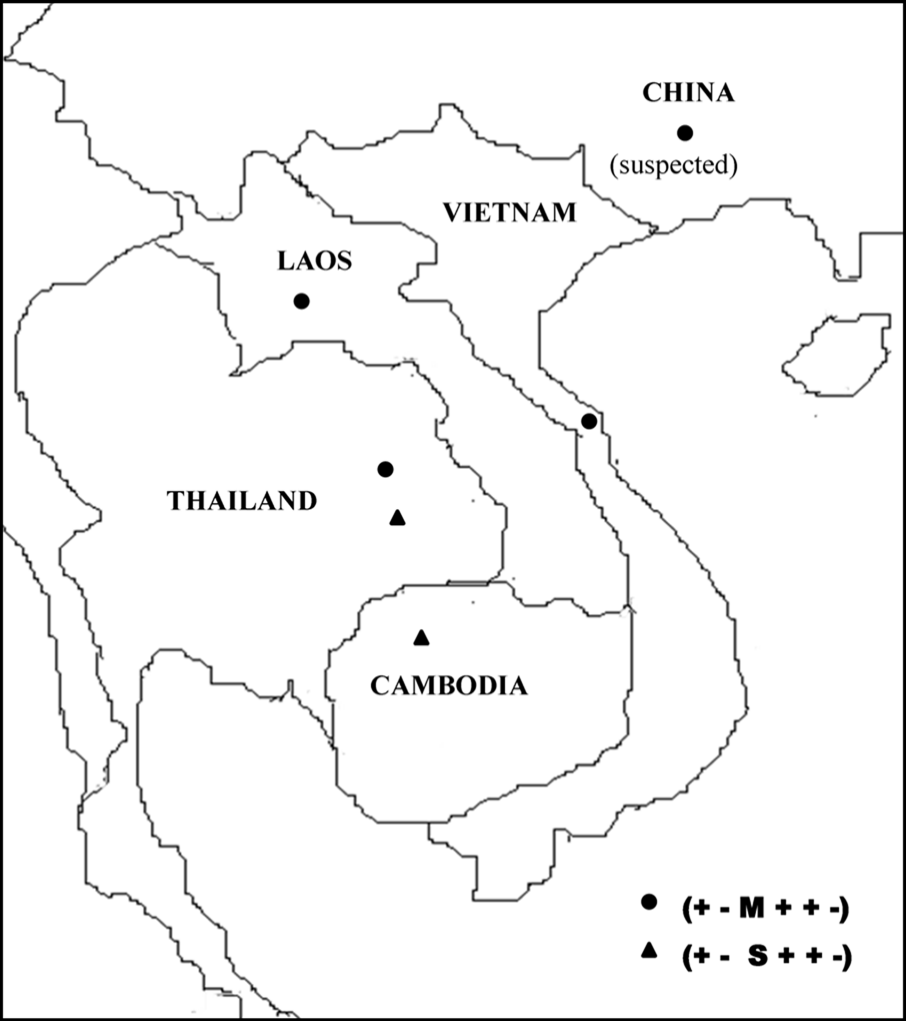
Map of Southeast Asia and China demonstrating distribution of the two major haplotypes associated with Hb CS alleles in the region.

**Fig 1**summarizes α-globin gene haplotypes associated with Hb CS genes in Vietnamese and Laotian subjects, as compared to those described in other Southeast Asian [10], Chinese and Mediterranean populations [9], although data for the latter two populations are relatively incomplete. Since no specimen from the family members of the subjects was available, only those of Hb CS linked haplotypes which could be segregated correctly were listed. These included 38 chromosomes of Vietnamese and 14 chromosomes of Laotian subjects. As shown in the Fig 1, 37 of 38 (97.4%) Vietnamese Hb CS genes were linked to haplotype (+—M + + -) and the remaining allele (2.6%) was associated with haplotype (-—M + + -). Among 14 Laotian Hb CS genes, 10 (71.4%) were linked to haplotype (+—M + + -). The remaining alleles were associated with 3 rarer haplotypes; (-—M + + -) (n = 1), (+—S + + -) (n = 2) and (+—S +—-) (n = 1). In contrast, haplotype (+—S + + -) is the major haplotype of Hb CS alleles in Thai and Cambodian populations [10]. We did not observe differences in hematological phenotypes among subjects with the same genotype but having different haplotypes. As summarized in the Fig 1, although a single Chinese Hb CS associated haplotype could not be defined for certain, it is most likely associated with the haplotype (+—M + + -), whereas the Mediterranean Hb CS alleles are linked to a different haplotype [9]. As for the Chinese, due to the lack of data on *Bgl* I, *Ac*c I and α*Pst* I polymorphic sites, the Mediterranean Hb CS haplotype could not be determined exactly. However, comparing with haplotypes described for the Mediterranean population [17], it is speculated that the Mediterranean Hb CS allele is associated with the haplotype IIa i.e. (-—L +—-), an undescribed haplotype in Asian populations, but presented at around 20% among the Mediterranean population. Based on these findings, it is apparent that there are at least three major haplotypes associated with Hb CS in these populations. As shown in **Fig 2**, two of them, (+—M + + -) and (+—S + + -), are found among Southeast Asian and Chinese, whereas the (-—L +—-) is detected only in the Mediterranean population.

**Fig 3:**
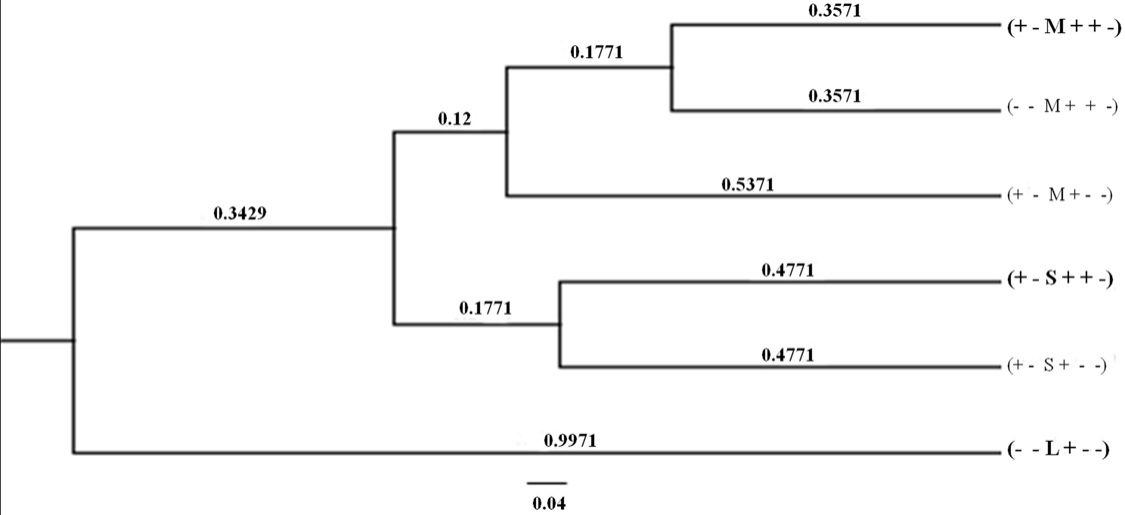
Phylogenetic tree of the Hb CS associated haplotypes constructed using the DendroUPGMA software applying Jaccard (Tanimoto) coefficient with default settings and 100 bootstrap replicates. Numbers indicate the branch lengths (divergence). Two major Asian Hb CS haplotypes; (+—M + + -) and (+—S + + -) and the predicted Mediterranean haplotype (-—L +—-) are indicated in bold face. The major haplotypes, (+—M + + -) and (+—S + + -), of Hb CS in Asian populations show common ancestors and are separated into each branch. In contrast, the Mediterranean haplotype represents clearly different origin.

**Fig 3**demonstrates a phylogenetic tree constructed with haplotypes observed using the DendroUPGMA software, with Jaccard coefficient and 100 bootstrap replicates, with the Cophenetic correlation coefficient (CP) of 0.79. As shown in the figure, it was found on the basis of branching that the two Asian Hb CS haplotypes; (+—M + + -) and (+—S + + -) share common ancestors and are separated into branches whereas the predicted Mediterranean haplotype (-—L +—-) represents a clearly different origin.

## Discussion

Hb CS [α2-globin gene, codon 142 *T*AA → *C*AA] appears to be the most prevalent non-deletional α-thalassemia in Asian. It has an incidence of 1–8% in the general Thai population and in Lao-speaking populations of the Mekong river basin in northeastern Thailand and the Lao PDR [18, 19]. The highest frequency of Hb CS of around 25% has been recorded in the Có-Tu ethnic group living in the mountainous areas of Thua Thien Hue, central Vietnam [8]. It has been found in other populations with lower frequencies e.g. Greek, Sicilian and United Arab Emirate families [20, 21]. This elongated α globin chain variant does not appear to be unstable but observations have suggested that α^CS^ mRNA is unstable, possibly due to inappropriate translation of the 3’non-coding region wherein the disrupted sequences are located [22]. It has been shown that binding of the α^CS^ globin chain on the inner membrane of red blood cell containing Hb CS can cause increased hemolysis and unusual pathobiology of red blood cells of the patient [23]. Heterozygous Hb CS is usually asymptomatic, but homozygous is characterized by overt hemolytic anemia and absence of microcytosis [2]. The hematological findings shown in Table 1 for various Hb CS genotypes confirm this. We observed a relatively normal hematological feature for Hb CS heterozygote (n = 89) and double heterozygote for Hb CS/Hb E (n = 23) although the latter would have less globin chain imbalance. Relatively mild anemia with Hb at 9.9±0.8 g/dl and MCV of 82.8±8.5 fl and lower Hb A_2_ level (1.4 ± 0.2%) as compared to heterozygous Hb CS (2.1 ± 0.2%) were observed for 9 cases with homozygous Hb CS. It has been recommended to maintain a high index of suspicion for Hb CS in a patient with mild anemia, no microcytosis and Hb A_2_ below 2%, before accurate diagnosis can be established by molecular testing [24]. No ameliorating effect of Hb CS on the hematological phenotype of a case with Hb E-β-thalassemia (α^CS^ α/ αα;β^0^/β^E^) was apparently observed. It is noteworthy that the hematological data of the two cases with Hb H-CS disease differ dramatically. This might be due to increased hemolysis secondary to infection or fever, which is commonly found in a non-deletional Hb H disease [25]. Since all of these thalassemias are very common in the region, identification of Hb CS is therefore important in routine practice. An Hb CS carrier may be missed during Hb screening, because the level of Hb CS is less than 1% unless DNA analysis is performed.

Of interest is the finding of a single haplotype (+—M + + -) (37 of 38 chromosomes examined) linked to Hb CS genes in the Có-Tu ethnic minority of Vietnam (Fig 1). Although a single Hb CS gene was associated with haplotype (-—M + + -), the phylogenetic tree shown in Fig 3 indicates that it shares the same origin with that of the former haplotype and they could be derived from each other by a single genetic recombination event. Based on this finding, the high frequency of Hb CS in this ethnic minority could not be explained by multiple origins of the Hb CS mutation in this population. This makes natural selection or frequent consanguineous marriage a more likely appropriate explanation. A remarkably high frequency of Hb CS in this minority of the Vietnamese population might be attributed to strong selection in favor of Hb CS during the malarious past. In fact, the remote habitat of this minority group in central Vietnam is still endemic for malaria [26]. Although no *in vitro* experiment or epidemiological work has suggested that Hb CS confers resistance against malaria, this has been observed in other forms of thalassemia and hemoglobinopathies including Hb S, Hb E and Hb C [17]. It has also been shown that α^+^-thalassemia deletion is associated with reduced cytoadherence of *Plasmodium falciparum* infected erythrocytes to microvascular endothelial cells and monocytes, and reduced risk of severe malaria [27].

In contrast, we found as many as 4 haplotypes linked to Hb CS genes in Laotian subjects among 14 chromosomes examined. As for the Vietnamese, the most prevalent one (10 of 14 chromosomes) is (+—M + + -). Taking all these findings and those described previously in Thai and Cambodian populations [10] into consideration, it is conceivable that two major haplotypes of Hb CS in the regions include (+—M + + -) and (+—S + + -), which are separated into different branches on the phylogenetic tree shown in Fig 3. While the former haplotype is apparently common among the northern part of the region, including northern Laos, Vietnam and south China, the latter is prevalent in the southern part of the region including northeast Thailand and Cambodia (Fig 2). Since, these two Asian haplotypes are identical, with the exception of the size of the inter-ζ hypervariable region i.e. M and S alleles, they could be derived from each other by a mutation event in this hypervariable region. However, the high frequencies of both haplotypes among Asian populations (Fig 1) likely suggests independent origin of Hb CS in Southeast Asia. This consideration provides at least a potential explanation for a high prevalence of Hb CS in the region. A similar pattern of haplotypic heterogeneity has been observed for Hb E, the most common Hb variant in Southeast Asia [28]. Nonetheless, as shown in Fig 1 and Fig 3, it is most likely that the Mediterranean Hb CS haplotype [9] has originated independently. The presumably Mediterranean haplotype (-—L +—-) separates into another main branch of the phylogenetic tree. Accordingly, the inter-ζ HVR polymorphism (S, M, L) seems to be a useful marker for differentiation of Asian Hb CS (S & M alleles) and Mediterranean Hb CS (L allele). These results indicate that Hb CS arose independently and more than once in the world population.
